# Homeopathy for seasonal allergic rhinitis: rationale, design and methods of the three-armed randomized controlled HOMEOSAR trial

**DOI:** 10.1186/s12906-022-03820-w

**Published:** 2022-12-22

**Authors:** J. Siewert, M. Teut, K. Gaertner, S. Binting, C. Eberhardt, M. Ortiz, W. Grabowska, T. Reinhold, S. Roll, B. Stoeckigt, S. N. Willich, H. Cramer, B. Brinkhaus

**Affiliations:** 1 Institute of Social Medicine, Epidemiology and Health Economics, Charité – Universitätsmedizin Berlin, corporate member of Freie Universität Berlin and Humboldt-Universität zu Berlin, Berlin, Germany; 2 Institute for Integrative Medicine, University of Witten/Herdecke, Herdecke, Germany; 3 Pharmacy Department, Freie Universität Berlin and Humboldt-Universität zu Berlin, Berlin, Germany; 4 Department of Internal and Integrative Medicine, Faculty of Medicine, Evang. Kliniken Essen-Mitte, University of Duisburg-Essen, Essen, Germany

**Keywords:** Homeopathy, Complementary and alternative medicine, Allergy, Seasonal allergic rhinitis, Randomized controlled trial

## Abstract

**Background:**

Patients with seasonal allergic rhinitis (SAR) frequently use homeopathic therapy. Although there is some evidence that homeopathy may be effective in treating symptoms of SAR, there is a lack of high-quality clinical trials. Therefore, the aim of the homeopathy for SAR (HOMEOSAR) trial is to determine the efficacy of individualized or standardized homeopathic drug treatment compared to placebo regarding rhinitis-related quality of life in patients with SAR.

**Methods:**

This randomized, placebo-controlled, double-blind, three-armed intervention study will be conducted at two university hospital outpatient clinics for complementary and integrative medicine in Berlin and in 12 office-based practices specializing in homeopathic treatment in Germany. A total of 270 patients with clinical symptoms of SAR and positive allergy test to birch and grass pollen will receive homeopathic anamnesis and subsequently be randomized into (a) standardized homeopathic drug treatment with Galphimia Glauca (potency D6), (b) individualized homeopathic drug treatment (D6), or (c) placebo. All three groups can receive on-demand rescue medication as needed. Treatment will consist of two consultations and daily intake of the study medication for 4 weeks during the pollen season. The primary outcome is the mean overall score of the Rhinitis Quality of Life Questionnaire (RQLQ) in weeks 3 and 4, analyzed using analysis of covariance (adjusted for baseline RQLQ overall score and study center). A closed testing procedure will be used to control the overall type I error comparing the 3 treatment groups. Secondary outcomes include the overall RQLQ and its seven domain scores, responder status (decrease in RQLQ overall score of at least 0.5 points compared to the baseline value), use of rescue medication, intensity of total and individual SAR symptoms based on visual analog scale, generic health-related quality of life, safety, utilization of health care resources and associated costs. In addition, a qualitative data analysis is planned.

**Conclusion:**

The results of our study will contribute to clarifying the possible therapeutic effects of homeopathic drug treatment for patients with SAR.

**Trial registration:**

This study has been registered in the German Clinical Trial Registry with trial ID DRKS00018081 on June 09, 2020.

## Background

Seasonal and perennial allergic rhinitis (AR) is a global health problem with a prevalence between 10 and 40% worldwide, affecting 2% to 25% of children and up to 40% of adults [1]. In Western Europe, the yearly prevalence of AR is 23% [2]. For Europe as a whole, the direct costs of AR have been estimated at 1.0 to 1.5 billion Euro, and its indirect costs have been estimated at 1.0 to 2.0 Euro billion annually [3].

Specific immunotherapy, the only causal treatment currently approved for seasonal allergic rhinitis (SAR), is time-consuming, costly, and not effective in all SAR patients [4]. Other available pharmacological treatments aiming to reduce the intensity of symptoms do not always achieve clinically relevant reductions and may cause considerable side effects. Consequently, a large number of AR patients turn to complementary medical treatments, particularly homeopathy [5–7].

In an observational study of 3,981 patients conducted in Germany and Switzerland, AR was one of the most frequent reasons why patients sought homeopathic treatment and the most common diagnosis in men who underwent classical homeopathic therapy [8].

In several trials, homeopathy has been implemented in the treatment of AR [9–17]; however, the evidence of its efficacy remains unclear. The best evidence for the efficacy of acute AR treatment is available for the homeopathic drug Galphimia glauca in decimal- (D-) potencies. A meta-analysis that included 11 studies with more than 1,000 patients demonstrated a stable therapeutic effect and a statistically significant superiority of Galphimia glauca over placebo. The clinical effect was described by the authors as being comparable to that of conventional antihistamines. Of note, all studies in the meta-analysis were conducted by the same research group [18, 19].

The most common homeopathic treatment strategy is individualized classical homeopathy. To date, the only studies that have investigated the efficacy of this treatment strategy for AR have been observational, with a high risk of selection bias, rather than randomized and placebo controlled. Nonetheless, a prospective observational study of 40 patients with atopic diseases, including AR, both seasonal and perennial allergic conjunctivitis, asthma, and neurodermatitis found clinically relevant benefits from individualized homeopathy in a prepost comparison. Furthermore, treatment satisfaction was rated as very high, and all but one patient desired to continue the therapy [17]. In another observational study with 46 patients, the prepost comparison of the disease-specific quality of life also suggested a clear improvement of SAR symptoms when treated with individual homeopathy [11].

In 2017, a systematic review with meta-analysis of studies on homeopathy in the treatment of seasonal or perennial AR was published [18, 19]. Of the 11 included studies, eight had a high risk of bias according to the Cochrane criteria. Only three studies using Galphimia glauca as an intervention were included in the meta-analysis. Regarding nasal and ocular symptoms, these studies showed efficacy in favor of homeopathy compared to placebo [20]. The authors of the systematic review concluded that, due to the heterogeneous methodological quality of the studies, any definitive assessment of homeopathic drug efficacy is questionable. They indicated that well-conducted randomized controlled trials (RCTs), which surpass identified methodological problems, are strongly required before definite conclusions on the efficacy of homeopathy for SAR can be drawn. Given the limitations of the evidence and the weak study methodology of hitherto published studies, homeopathy is not included in current national German treatment guidelines for AR. Indeed, the current AWMF (the Association of the Scientific Medical Societies in Germany) guideline states that “the effectiveness of homeopathy cannot be conclusively assessed due to the limited data available” [21]. Given that the effectiveness of homeopathy for AR has not been studied exhaustively yet, further research adhering to the highest methodological standards is needed.

### Aims

We aim to assess the efficacy of a 4-week course of i) standardized drug treatment with homeopathic preparations of Galphimia glauca or ii) an individualized homeopathic low potency drug compared to placebo with regard to disease-specific quality of life in patients with SAR.

## Methods

### Study design

**Fig. 1:**
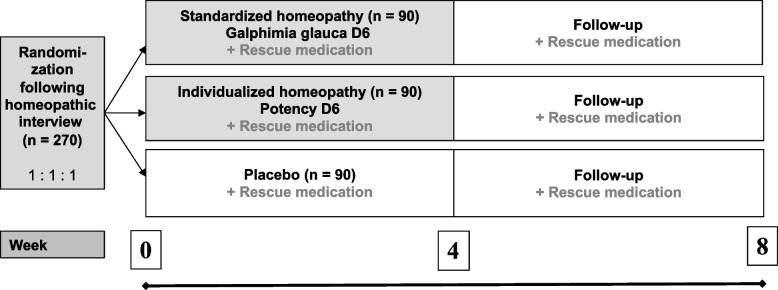
Design of the HOMEOSAR study. Abbreviations: *D*  Decimal potency. On-demand rescue medication consists of the oral antihistamine cetirizine (10 or 20 mg) plus, or only, if necessary, oral cortisone

HOMEOSAR (Homeopathy for Seasonal Allergic Rhinitis**)** is a three-armed, phase IV, randomized, parallel group, placebo-controlled, double blind, multicenter interventional trial for patients suffering from SAR. The study will be conducted for 8 weeks per patient, including a treatment phase of 4 weeks and a follow-up observational phase of 4 weeks (Fig. 1). By including a qualitative component, the study will follow a mixed methods approach. The Recommendations for Interventional Trials (SPIRIT) 2013 [22, 23] were also followed in the design of this trial.

### Patients and recruitment

A total of 270 patients aged 18 to 75 years and diagnosed with moderate to severe SAR will be included in the trial. The inclusion criteria consist of a skin prick test and/or a Radio-Allergo-Sorbent Test (RAST) positive for both birch and grass pollen, a score between 40 and 80 mm on a visual analog scale (VAS) for the average intensity of SAR symptoms in the week prior to inclusion, an indication for the use of oral antihistamines as anti-allergic medication, and written informed consent.

The exclusion criteria include perennial allergic rhinitis or other types of chronic rhinitis; history of anaphylactic reactions; moderate to severe atopic dermatitis; and symptoms of asthma in accordance with the GINA criteria with more than one of the following: (1) occurrence of asthma symptoms more than twice per week, (2) any night waking due to asthma, (3) reliever (beta-2-sympathomimetic drug) needed more than twice per week, except for reliever taken before exercise, (4) activity limitation due to asthma. Further exclusion criteria are renal insufficiency (serum creatinine concentration above 0.6–1.4 mg/dl or 50–130 μmol/l) measured within the last 16 weeks; current daily use of topical or systemic corticosteroids; known hypersensitivity to Galphimia glauca or other homeopathic drugs; allergen-specific immunotherapy used currently or within the past 3 years; homeopathic therapy used currently or within the past 6 weeks; a current or recent (the past 6 weeks) use of other complementary and integrative medicine treatments for SAR; alcohol addiction; pregnancy or breastfeeding; serious acute or chronic organic diseases or serious mental disorder; an acute infection with SARS-CoV-2 virus, long-COVID-syndrome, or a positive SARS-CoV-2 PCR or Rapid Antigen Test obtained 24 h prior to the baseline and/or at the 2-week follow-up visit.

For the qualitative part of the study, semistructured interviews with about 15 randomly selected study participants will be conducted.

The recruitment of study participants will be carried out primarily by advertising on public transport, in newspapers, and through flyers at general practitioners’ clinics.

### Study physicians

This multicenter RCT will be conducted at two outpatient clinics for complementary and integrative medicine located at the university hospital in Berlin (Charité – Universitätsmedizin Berlin) and at 12 family medicine clinics specialized in homeopathic treatment located in the Berlin and Munich metropolitan areas, Germany. The study physicians are medical doctors required to hold certifications for medical specialists, e.g., general medicine (German: “Facharztbezeichnung”) and an additional qualification for homeopathy (German: “Zusatzbezeichnung Homöopathie”), as well as at least five years of practical experience in providing homeopathic treatment.

### Study procedures

The study will be conducted during the birch and/or grass pollen season (this is usually between February and the end of July in Germany). After providing written informed consent, all patients included in the study will receive a baseline assessment, rescue medication, and subsequently a first homeopathic consultation of 60 to 120 min. After the consultation, study physicians will prescribe an individualized homeopathic drug. The prescription will then be sent by fax to the study pharmacy, where the patient will be randomized into one of the two homeopathic drug treatment groups and the placebo group and obtain his or her allocated study medication for the duration of the study.

In case of need, patients in all groups are allowed to take predefined rescue medication and will be instructed to document the daily applied rescue medication in the medication diary between baseline and the end of week 4, as well as in weeks 7 and 8. Furthermore, the dosage of the study medication will be documented daily between baseline and the end of week 4. Additional patient questionnaires will be distributed to patients at baseline and sent directly to patients by the study administration center at the end of weeks 2, 4, and 8. These questionnaires will consist of the Rhinitis Quality of Life Questionnaire (RQLQ); a visual analog scale measuring symptom intensity of SAR; a questionnaire on generic health-related quality of life (SF-12); as well as questions concerning the severity of asthma and atopic eczema, health care resource utilization, and expectations and assumptions regarding treatment group allocation.

After the 4-week treatment phase, the patient will be instructed to hand any remaining study medication and the original packets of the rescue medication back to the investigators in a pre-postaged return envelope.

### Intervention and rescue medication

After randomization by the study pharmacy, patients will receive either the registered homeopathic drug Galphimia glauca in potency D6, an individualized and registered homeopathic drug selected by the study physician in potency D6, or the placebo treatment. All patients will receive their study medication as alcoholic dilution in identical bottles and start treatment within 5 days after the first consultation. The standard dose is five drops of the assigned study drug taken orally three times a day. The prescribed drug dosage can be individually adjusted (i.e., increased or decreased) by the study physician, in accordance with the established homeopathic treatment principles. Patients will also be advised to store the medication in a dark place at room temperature, separate from other drugs, and to shake the bottle strongly ten times according to homeopathic recommendations prior to taking the medication. The second homeopathic consultation (duration of a maximum of 30 min) will be conducted for all patients approximately 14 days after the first homeopathic consultation. Although study physicians may prescribe another individualized homeopathic drug to each patient after two weeks, only those randomized to the individualized homeopathic treatment group will receive the changed homeopathic drug. Patients in the other two treatment groups will continue to receive standardized treatment (Galphimia glauca D6) or placebo.

Galphimia glauca D6 and all individualized homeopathic products with a potency of D6 are registered and routinely used medicinal products in Germany. They are available as “over the counter” drugs in any German pharmacy and are registered by a positive monograph from Commission D (German: Kommission D) of the Federal Institute for Drugs and Medical Devices (BfArM). In the individualized homeopathic drug treatment arm, any homeopathic medicinal product that is registered, monographed, and available from “Deutsche Homöopathie-Union” (manufacturers of homeopathic medicinal products) can be prescribed.

Patients in all treatment groups may take second-generation oral antihistamines (10 or 20 mg cetirizine) on demand as rescue medication. In case when a daily maximum dose of 20 mg cetirizine is not sufficiently effective, patients may additionally take an oral cortisone preparation (max. prednisolone 10 mg/day). During the 8-week study period, patients are asked not to use any concomitant homeopathic treatment or any other complementary and integrative medical treatments (e.g., herbal medicine, acupuncture, and others) for the treatment of SAR symptoms.

### Outcome measures

**Fig. 2:**
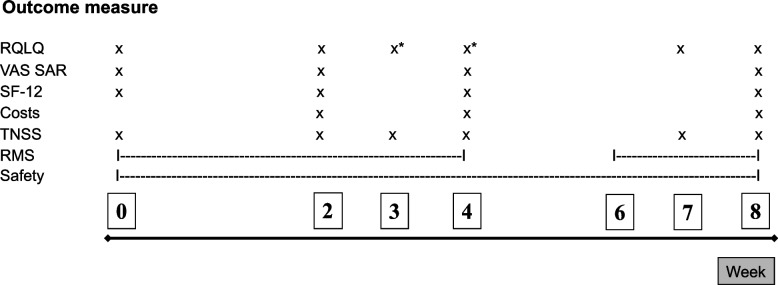
Data collection time points of primary and secondary outcome measures. Abbreviations: *RQLQ*  Rhinitis Quality of Life Questionnaire, *VAS SAR*  Visual analog scale of seasonal allergic rhinitis symptoms, *SF-12*  Generic health-related quality of life questionnaire Short Form 12, *TNSS*  Total Nasal Symptom Score, *RMS*  Rescue Medication Score. * Primary outcome: Mean of the two RQLQ overall scores

**Fig. 3:**
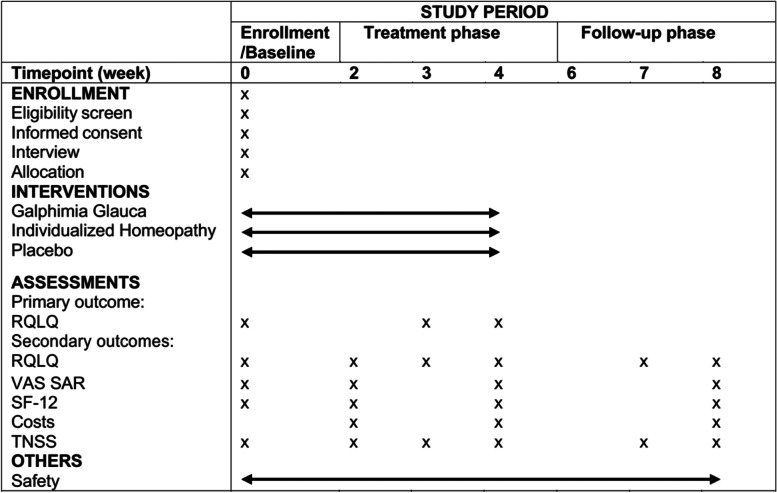
Standard Protocol Items: Recommendations for Interventional Trials (SPIRIT) Figure. Abbreviations: *RQLQ*  Rhinitis Quality of Life Questionnaire, *VAS SAR*  Visual analog scale of seasonal allergic rhinitis symptoms, *SF-12*  Generic health-related quality of life questionnaire Short Form 12, *TNSS*  Total Nasal Symptom Score, *RMS*  Rescue Medication Scor e

The primary outcome is disease-related quality of life (RQLQ, Fig. 2). To allow enough time to assess the drug effect and to have a broad time window to compensate for weekly fluctuations in pollen flow and SAR symptoms, the RQLQ mean of weeks 3 and 4 was chosen as the primary outcome (Fig. 3).

The RQLQ is a well-established and validated questionnaire developed by Juniper et al. [24, 25]. It has been used successfully as a primary [15, 26–28] or secondary endpoint [29] in numerous studies of high methodological quality. The RQLQ consists of 28 questions in 7 domains, including activity limitation, sleep problems, nose symptoms, eye symptoms, other symptoms, practical problems, and emotional state, ranked from 0 (no impairment) to 6 (severe impairment). The RQLQ also captures nasal symptoms as a standard endpoint for allergic reactions [30].

Secondary outcome measures will be measured at the end of weeks 2, 3, 4, 7, and 8 and will include the RQLQ overall score and the seven RQLQ domain scores, nasal symptoms (Total Nasal Symptom Score, TNSS), the use of rescue medication (Rescue Medication Score, RMS), and SAR symptoms (nasal and nonnasal, 100 mm VAS ranging from 0 mm = no symptoms to 100 mm = max. symptoms). The responder status is defined by at least a 0.5-point decrease (improvement) in the RQLQ overall score (mean of weeks 3 and 4) compared to the baseline value. The generic health-related quality of life (SF-12) questionnaire will be used to assess the perceived homeopathic drug treatment effect on generic quality of life and to measure quality-adjusted life years (QALYs). For the health economic analysis, the patient-specific use of health care resources during the study (doctor contacts, hospital stays, medication, remedies and aids, work absence and incapacity days) will be documented continuously, and the associated costs will be calculated. Combined with the results on QALYs, a cost-effectiveness assessment of the study treatment will be carried out. Adverse events (AEs), serious adverse events (SAEs), and the number of patients who discontinue treatment due to AEs or SAEs will be collected from baseline until completion of the study.

In the qualitative part of the study, the subjective experiences of treatment, treatment satisfaction and subjectively perceived effects of the treatment will be assessed.

### Blinding procedures

The study participant, study physician, principal investigator, sponsor, study nurse, data manager and biometrician, as well as the qualitative researcher, will be blinded to treatment. To ensure blinding, the study pharmacy will produce placebo preparations that are, after labeling, indistinguishable from the study medication.

The possibility of unblinding in emergency situations (e.g., when necessitated by an SAE) is ensured during the study. Sealed emergency envelopes with the treatment assignments will be prepared by an independent data manager at the sponsor’s site, who is otherwise not involved in the study.

### Randomization

Patients will be randomized by block randomization with randomly varying block lengths, stratified by study center. Patients will be randomized into one of the three groups (standardized homeopathy, individualized homeopathy, or placebo) in a 1:1:1 ratio using “R” software (The R Foundation, Free Software Foundation, Version 3.6.1, Boston, USA). The randomization list will be generated by a biometrician at the sponsor’s site, who is otherwise not involved in the statistical analysis of the study and has no access to study data. The results of the randomization will be integrated into a database (Microsoft ACCESS 2016, Redmont, USA) by an independent data manager, who is also responsible for the creation of the emergency envelopes but is otherwise not involved in the study. The patient code and the group allocation will be generated by the pharmacy during randomization (in an ACCESS database). Based on this database, the study pharmacy will allocate the patients into one of the homeopathic drug treatment groups. The group allocation will be known only to the pharmacist and one independent unblinded data manager who is not further involved in the study organization. If a patient withdraws from the study, the randomization number assigned to them may not be assigned again; withdrawn patients will not be eligible for study re-entry at a later point in time.

### Sample Size calculation

The study is designed to detect a minimal clinically important difference (MCID) between either one of the two drugs and the placebo group with 80% power. For the RQLQ, the MCID is 0.5 points [25]. The common standard deviation, based on baseline data from an earlier study [27], is estimated at 1.1, corresponding to an effect size of d = 0.45. With these assumptions, *n* = 77 patients per treatment group are needed (i.e., a total of *n* = 231 patients in three treatment groups). To compensate for up to a 15% dropout rate [27], we plan to randomize *n* = 270 patients (i.e., *n* = 90 per treatment group).

### Statistical analysis

Analyses will be performed for 4 populations. (1) An intention-to-treat (ITT) population, including all randomized patients regardless of whether data are available, whether the intervention was performed according to the protocol, whether unauthorized concomitant interventions were undertaken, or whether any other protocol violations occurred. Every patient will be analyzed as if they had conducted the trial according to the group to which they were originally allocated. Missing values in primary and secondary outcomes will be imputed using multivariate imputation by chained equations (MICE). (2) A per-protocol population (PPP) will include only patients with no major protocol deviations. (3) A safety set, including all randomized patients who receive at least one dose of the study drug, will be analyzed according to the study drug they received. (4) A safety set without taking comedication, will include all patients who received at least one dose of their allocated drug and did not take rescue medication or any other medication during the study period and will be analyzed as treated.

Sociodemographic and medical baseline data will be analyzed descriptively in terms of the mean, standard deviation, median, minimum, and maximum for continuous data or in terms of frequencies and percentages for categorical data (for each treatment group and in total).

The primary endpoint is defined as the mean of the RQLQ total score at the end of weeks 3 and 4. A closed testing procedure [31] will be used for the primary outcome to handle the pairwise comparisons between 3 treatment groups to control the overall type I error and to maintain the global significance level of α = 5% (two-sided). First, a global test will be used across all 3 treatment groups to test the null hypothesis of equal means. If this null hypothesis is rejected, two pairwise comparisons (individual homeopathy vs. placebo, standardized homeopathy vs. placebo) will be tested confirmatively. The comparison of individualized homeopathy vs. standardized homeopathy will be tested exploratively. The analysis will be performed on the ITT population using an analysis of covariance (ANCOVA), in which the endpoint is modeled as a function of treatment group (fixed factor), baseline RQLQ score (linear covariate), and the study center (random factor) to obtain the treatment group means, mean treatment group differences, respective 95% confidence intervals, and the (two-sided) p values. All secondary endpoints will be analyzed exploratively using both the ITT and PPP analysis sets. Continuous secondary endpoints will be analyzed by ANCOVA, in which the endpoint is modeled as a function of treatment group (fixed factor), the baseline value of the respective variable (linear covariate), and the study center (random factor) to obtain the mean treatment group differences, the 95% confidence intervals, and the explorative p values (two-sided). Responder rates will be compared between treatment groups using logistic regression adjusted for the baseline RQLQ value and the study center (random factor).

Safety endpoints will be analyzed descriptively for the two safety sets.

The monetary evaluation of resource use will be undertaken using German standardized cost rates. The results will be tested for relevant and statistically significant differences as part of a cost comparison analysis between the treatment groups. Additionally, QALYs will be calculated based on the results of the SF-12 questionnaire and used for a cost-effectiveness analysis. In case of the superiority of a treatment group in terms of QALYs, the incremental costs per QALY gained (ICER) will be obtained by combining cost and effect differences between treatment groups. In the case of increased costs and improved quality of life, the ICER indicates the cost-effectiveness by adopting an internationally accepted threshold (e.g., ≤ 50.000 Euro per QALY gained). Besides QALYs, further outcomes, including RQLQ changes and number of responders, will be used for estimating the total treatment costs. All cost-effectiveness results will be visualized in a cost-effectiveness plane.

### Qualitative study part

The interviews for the qualitative data collection will be recorded digitally, transcribed verbatim using pseudonyms, and gradually coded, categorized, and analyzed using qualitative content analysis [32]. In the semistructured individual interviews, patients will be asked about subjective experiences of the treatment and their subjectively perceived effects of the therapy on their symptoms, well-being, and quality of life. With this mixed-method approach, the quantitative results of the study can be supplemented and expanded, while further possible effects can be presented in the form of hypotheses [33].

### Data monitoring and safety board

The Data Monitoring and Safety Board (DSMB) will include independent experts and will be established prior to the start of the study. It will ensure and monitor the safety of the trial participants, the credibility of the study, and the validity and integrity of the data. The DSMB is also responsible for evaluating SAE reports and assessing suspected unexpected serious adverse reactions (SUSARs). To determine the feasibility of recruitment, interim data reports will be submitted every month during the pollen season and every three months after the recruitment phase is completed. The DSMB will consist of a statistician, an expert for research in chronic diseases and complementary and alternative medicine, and a homeopathic family physician.

### Audit/inspection

At any time during the study, an audit may be carried out by the sponsor as part of its quality assurance system or an inspection by the competent authority.

### Data management

The personal data of the patients will be entered into a password protected and encrypted Microsoft ACCESS database. The transfer from the paper-based CRFs into the computer system will be implemented pseudonymously. The data manager will be blinded to the data until the final stage of analysis. The qualitative interview transcripts will be pseudonymized and coded.

### Confidentiality

Patient data will be documented and identified with code numbers in a pseudonymized form.

## Discussion

The aim of the study is to perform a randomized controlled double-blinded trial with a sufficient sample size to assess the efficacy of homeopathy, an individual homeopathy or Galphimia glauca, for patients with SAR. The HOMEOSAR trial is the first clinical study to investigate the efficacy of both individualized and standardized homeopathic drug treatment in patients with SAR compared to placebo. In contrast to the former RCTs on homeopathy, and in particular on Galphimia glauca, HOMEOSAR is a randomized, multicenter three-armed study that assesses the efficacy of homeopathy from a broader perspective and thereby offers a higher external validity.

Our requirement is that the inclusion and exclusion criteria are comparable to other high-quality studies on SAR, including our own studies, in particular the ACUSAR trial on acupuncture in SAR [27, 34]. The study inclusion and exclusion criteria are based on the Consensus Statement in AR [3]. Due to a very low enrollment (*n* = 4) in the first recruitment phase during the COVID-19 pandemic, we have adjusted the inclusion and exclusion criteria for the subsequent pollen season. The changes include dropping the class 2 requirements for pollen and birch tests, dropping the requirement for the presence of SAR symptoms in the year prior to the year of enrollment, extending the upper age limit from 65 to 75 years old, shortening exclusion by the use of other complementary and integrative medicine treatments for SAR from 3 and 6 months to 6 weeks prior to study enrollment, and adding COVID-19-related health status and testing requirements. We have also changed the exclusion criteria based on asthma diagnosis from “partly controlled or uncontrolled asthma” to the presence of asthma in accordance with the GINA criteria listed in the “Patients and Recruitment” section of this publication.

To guarantee that the recruitment phase lasts long enough and that the SAR symptoms persist consistently, the inclusion criteria comprise the simultaneous presence of grass and birch allergies. In Germany, the birch and pollen seasons last approximately 5 to 6 months. Thus, the inclusion of patients with birch and grass pollen allergies will enable the recruitment of patients over a period lasting from February until July in any given year. We are aware that there is a high variability of SAR symptoms during the grass and pollen season, caused by high fluctuations in the amount of pollen flow. However, we expect that randomization, together with an extended recruitment phase will ensure an equal distribution of symptom severity among patients.

The necessity for individual homeopathic prescriptions is a known obstacle for double-blind trials [35]. Correspondingly, the follow-up consultation under the double-blind consensus and the limited timeframe will be a challenge for the study physicians, and we are aware that finding an individualized drug may require more time in the daily homeopathic medical practices.

To date, the evidence regarding the efficacy of homeopathy for SAR has shown overall mixed results [7, 9, 11, 13–19, 36]. However, homeopathic drugs seem to provide a low-risk safety profile [37], a hypothesis that remains to be proven in larger observation trials.

By performing this trial, we hope to yield high-quality data on the efficacy of homeopathic drugs for SAR. The results of this study will make an important contribution regarding the question of whether individualized and nonindividualized homeopathic drugs truly have an effect on SAR and whether this intervention can be recommended for this condition.

### Trial status

The recruitment launched on 11 April 2021 with a second recruitment year in the pollen season 2022, followed by a third recruitment period in 2023. Participant recruitment and data collection are expected to be completed by the end of October 2023.

## Data Availability

The anonymized final datasets that will be generated in this study will be available from the corresponding author on reasonable request after the publication of the trial results.

## Abbreviations

AE: Adverse events
ANCOVA: Analysis of covariance
AR: Seasonal and perennial allergic rhinitis
D: Decimal potency
HOMEOSAR: Homeopathy for Seasonal Allergic Rhinitis
ITT: Intention-to-treat population
MCID: Minimal clinically important difference
PP: Per-protocol population
QALYs: Quality-adjusted life years
RAST: Radio-Allergo-Sorbent Test
RMS: Rescue Medication Score
RQLQ: Rhinitis Quality of Life Questionnaire
SAE: Serious Adverse Events
SF-12: Generic health-related quality of life questionnaire
SPIRIT: Standard Protocol Items: Recommendations for Interventional Trials
SUSARs: Suspected Unexpected Serious Adverse Reactions
TNSS: Total Nasal Symptom Score
VAS: Visual analog scale
VAS SAR: Visual analog scale of seasonal allergic rhinitis symptoms
